# MMDCP: Multi-Modal Dental Caries Prediction for Decision Support System Using Deep Learning

**DOI:** 10.3390/ijerph191710928

**Published:** 2022-09-01

**Authors:** Soualihou Ngnamsie Njimbouom, Kwonwoo Lee, Jeong-Dong Kim

**Affiliations:** 1 Department of Computer and Electronics Convergence Engineering, Sun Moon University, Asan 31460, Korea; 2 Genome-Based BioIT Convergence Institute, Sun Moon University, Asan 31460, Korea

**Keywords:** convolutional neural network, artificial neural network, multi-modalities, hybrid neural network, dental caries

## Abstract

In recent years, healthcare has gained unprecedented attention from researchers in the field of Human health science and technology. Oral health, a subdomain of healthcare described as being very complex, is threatened by diseases like dental caries, gum disease, oral cancer, etc. The critical point is to propose an identification mechanism to prevent the population from being affected by these diseases. The large amount of online data allows scholars to perform tremendous research on health conditions, specifically oral health. Regardless of the high-performing dental consultation tools available in current healthcare, computer-based technology has shown the ability to complete some tasks in less time and cost less than when using similar healthcare tools to perform the same type of work. Machine learning has displayed a wide variety of advantages in oral healthcare, such as predicting dental caries in the population. Compared to the standard dental caries prediction previously proposed, this work emphasizes the importance of using multiple data sources, referred to as multi-modality, to extract more features and obtain accurate performances. The proposed prediction model constructed using multi-modal data demonstrated promising performances with an accuracy of 90%, F1-score of 89%, a recall of 90%, and a precision of 89%.

## 1. Introduction

Dental caries is one of the most highly chronic oral disease affecting populations of various ages worldwide [1,2] and was reported in 2010 as the 10th most prevalent Condition. According to the World Health Organization (WHO), approximately around 1.8 billion caries cases are recorded every year.

Nowadays, dental caries has become a problem for oral health and healthcare in general. Despite the action taken by current healthcare, the speculated reasons for the high recurrence of caries are suspected to be poor oral health behaviors and failure to attend appointments due to financial situation [3]. However, the current works to tackle the dental caries issue are either restrained or insufficient. Saliva, hereditary, bacteria and diet are some factors leading to dental caries. The authors of [4,5] showed how bacteria from diet caused dental caries in some patients. For example, sugar (sweet snacks), forming acids on the tooth’s surface [6], is one of the commonly consumed products. Two methods are used to diagnose dental caries: radiographic procedures, consisting of detecting dental caries using digital X-ray images [7]; and clinical method, which is classified as a visual examination of oral condition to detect caries [8]. These works have one thing in common, the usage of only one type of data (either image data or clinically collected data) for dental caries detection. Meanwhile, combining several types of oral healthcare data could generate a more credible diagnosis, therein removing the uncertainty of the possible sources of the disease. This uncertainty can cause the ratio of dental caries population to increase drastically. Given the growing number of people being affected by dental caries and the deficiency in methods to preclude more people from being affected by dental caries, a solution to tackle that deficiency was highly needed. Therefore, a Decision Support System (DSS) that can accurately provide insight into dental caries is needed to prevent more people from dental caries. Machine Learning (ML) has been used in recent years to help improve a diverse DSP system. Some works were conducted in oral health using ML, and several prediction algorithms have been applied to detect dental caries. Support Vector Machines (SVM) were used to classify dental root caries [9]; Random Forest (RF) and Artificial Neural Network (ANN) were used and provided good results in detecting dental caries [10].

The main contribution of this work is to propose a novel approach for dental caries prediction using a multi-modal deep neural network with two pathways and having the merit of learning patterns from heterogeneous features of the different data sources and making accurate predictions. Considering the ability of multi-modal to provide more information using different data sources, we utilized the Hybrid model (consisting of Convolutional Neural Networks (CNN) and Artificial Neural Networks (ANN)) to learn from the gathered data to predict dental caries precisely and accurately within a given population.

The remainder of the proposed work is organized as follows: Section 2 presents related works about ML, multi modalities, dental caries prediction, and prediction using Hybrid model. This section presents papers using multi-modality similar to the proposed model. Section 3 explains the proposed prediction method using the multi-modal data based on a hybrid neural network. Experimentation is described in Section 4. The results of this study and discussion are presented in Section 5. Finally, Section 6 concludes the paper.

## 2. Related Work

Artificial Intelligence (AI) has been used in every work domain for the past few years. They are extensively being used in the medical healthcare domain to better people’s lives. Given the fast development witnessed in the AI field, together with a large amount of healthcare data available, researchers are opting more for computational prevention approaches to develop new tools that accurately and efficiently diagnose patients [11]. Dietary habits and oral hygiene are essential in achieving and maintaining overall emotional and physical well-being throughout life [12]. Some improvements were observed in oral healthcare with the application of Machine learning to the dental dataset. Dental caries in oral healthcare underwent extensive experiments, and several prediction methods have been proposed in recent years. Kang, I.-A. et al. [10] proposed a dental caries prediction based on survey data from the Korean Center for Disease Control and Prevention under the Ministry of Health and Welfare. In this work, the authors have experimented on seven different ML algorithms (Random Forest (RF), ANN, CNN, Gradient Boost Decision Tree (GBDT), Support Vector Machines (SVM), Logistic Regression (LR), and Long Short-Term Memory (LSTM)). RF displayed the highest performance in predicted dental caries out of the seven ML used. Hung, M. et al. [9] presented a work on the classification of the presence or absence of root caries based on public data from the National Center for Health Statistics in the United States. Out of the algorithms used, SVM generated the highest performance during evaluation. Liu, L. et al. [13] constructed a generalized regression neural network (GRNN) model to predict caries among residents of Liaoning, China. Using regression on some highly correlated data tends to cause spurious results and overfitting [14].

Healthcare has exploited the advantage of automatically extracting essential features from the image dataset offered by the Deep Learning (DL) models [15]. CNN was one of the most used algorithms to detect, segment and classify organ-related diseases in medical image data [15,16,17]. DL has also been widely used in Dental caries classification and prediction. The authors of [18,19] presented a review study on early detection and diagnosis of dental caries on periapical radiographs using CNN. The authors of [20] proposed a CNN algorithm for dental caries prediction and two types of proximal and occlusal caries, showing respectively 85.6% and 83.6% accuracy. Zanella-Calzada, L.A. et al. [21] proposed a deep ANN for classifying subjects with caries and those without caries based on their dietary factor, demography, or nutritional feature (food elements, energy, and nutrients). The study [22] developed a CNN model for caries detection on bitewing radiographs using U-shaped deep CNN (U-Net). Dentist professionals are facing challenges in accurate diagnosis of deep caries and pulpitis on periapical radiographs; therefore, Zheng, L. et al. [23] experimented on CNNs (VGG19, ResNet18 and Inception V3) and ResNet18 + C (integrated with clinical parameter) for the diagnosis of deep caries and pulpitis displayed the highest performance with an accuracy of 82%. Zhang, X. et al. [24] Developed a DL model named ConvNet by adapting from Single Shot MultiBox Detector; the proposed model was trained automatically and was evaluated for classifying the presence of dental caries from photograph data. The developed algorithm displayed high performance, with an Area Under the Receiver Operating Characteristic Curve (AUC-ROC) of 85.65%. The model achieved a box-wise sensitivity of 64.60% and image-wise sensitivity of 81.90% at a high-sensitivity operating point. Various strategies were used to mitigate issues related to class imbalance, overfitting, and training data scarcity, and a simplified binary task for absence or presence of carious lesions was evaluated from regions of interest using the CNN model. The model achieved an AUC-ROC of 85.6% and 83.6% for proximal lesions and occlusal, respectively [21].

In recent works, Multi-modal has been shown promising results and considered by several researchers. Multi-modal in ML is a multi-disciplinary field that integrates and models multiple modalities, including visual messages, linguistics, and acoustic, and therefore addresses some of the original goals of AI. Healthcare researchers have made use of the advantages displayed by multi-modality. Li, W. et al. [25] published a survey on emotion recognition using bio-signal data (EDA, EMG, RESP, ECG, TEMP) as input data. The authors of [26,27,28] proposed a multi-modal architecture for stress level recognition using multi-modal data. The authors of [29] classify, detect and estimate affective state ahead of time using physiological and motion data based on a hybrid model (a CNN used to predict the signal value in time step *t* + 1 and a RF used to predict the affective state). Venugopalan, J. et al. [30] proposed a Multi-modal DL model for early detection of stages of Alzheimer’s disease, patients were classified into three categories, AD, MCI and controls (CN). The authors Trained the DL model with three types of data during this experiment: clinical test data, genetic (single nucleotide polymorphisms (SNPs)) and imaging (magnetic resonance imaging (MRI)); the multi-modality aspect of their approach provided a holistic view of AD staging analysis. Pingali, L. [31] proposed multi-modal ML-based knee osteoarthritis (OS) progression prediction from clinical data and plain radiographs. The proposed approach could improve selecting the appropriate patient to receive help for developing personalized therapeutic plans and OA drug-development trials. Tiulpin, A. et al. [32] proposed a 3D DL model that automatically extracts features from multi-modal preoperative brain images (T1 MRI, FMRI and DTI) to predict if a patient has a short or long overall survival time from brain tumor disease. The proposed project provides users with real-time views of sensed data, collects health information, stores tagged sensor data in a central cloud database and shows classification results and advice. The author of [33] developed a personal, multi-modal, and cost-effective Oral Health Advisor that automatically classifies sensed data and provides interactive advice about oral health. From the research mentioned above, multi-modal ML has shown the advantage of experimenting with several data sources and having more accurate estimation due to the significant number of aggregated features present in the fusing data used. A few oral healthcare research for dental caries prediction using multi-modalities have been carried out. Our proposed work comes as a novel approach for dental caries prediction using a hybrid ML algorithm fed with Multi-modal data.

## 3. Proposed Model

**Figure 1:**
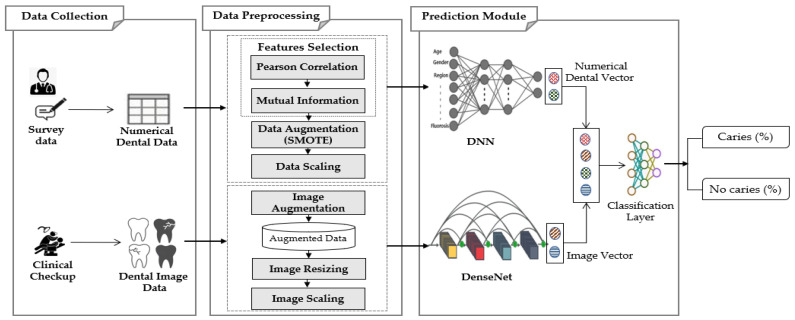
Proposed multi-modal dental caries prediction model.

This work proposes the MMDCP (Multi-Modal Dental Caries Prediction) model, consisting of 3 stages: data collection, data preprocessing, and prediction module. The model uses a multi-modal dataset obtained from different sources. The two datasets are separately preprocessed at the preprocessing stage, then a hybrid algorithm (Composed of ANN and DenseNet201) is used on the preprocessed data. The two neural networks were fused to learn from the cross-modality joint heterogeneous features from the dataset. Figure 1 schematically illustrates the proposed MMDCP model.

### 3.1. Data Collection

Similar works in the field of dental health problems prediction use either numerical datasets or image type of datasets to train their models. The size and diversity of the datasets allow the model to have sufficient parameters to detect hidden abnormalities present in patients tooth. Hence, this work uses two types of datasets; the first dataset is from a children’s oral health consultation survey conducted by medical doctors in 2018 and made publicly available by the Korean Center for Disease Control and Prevention under the Ministry of Health and Welfare. The dataset contains 22,371 samples divided into two groups (patient without dental caries (18,954), and patient with caries (3417)). The dataset consists of features like age, gender, bleeding gum, dental history, etc. The second dataset consists of teeth images collected from different online sources [34,35] with a total of 2592 images samples, these samples were divided into two classes (teeth without caries (1754), and teeth with caries (839)).

### 3.2. Data Preprocessing

As mentioned in the data collection stage, the proposed algorithm uses two types of data, a numerical dataset having 90 features and 22,371 samples, and an image dataset with a total of 2592 samples. These datasets must undergo through a series of preprocessing steps before being used by the proposed hybrid model to display high performance.

#### 3.2.1. Numerical Dataset Preparation

**Figure 2:**
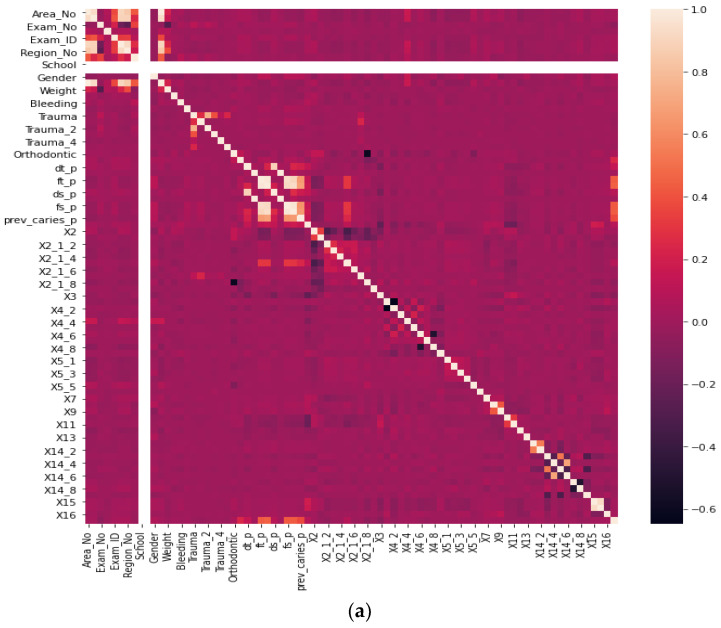

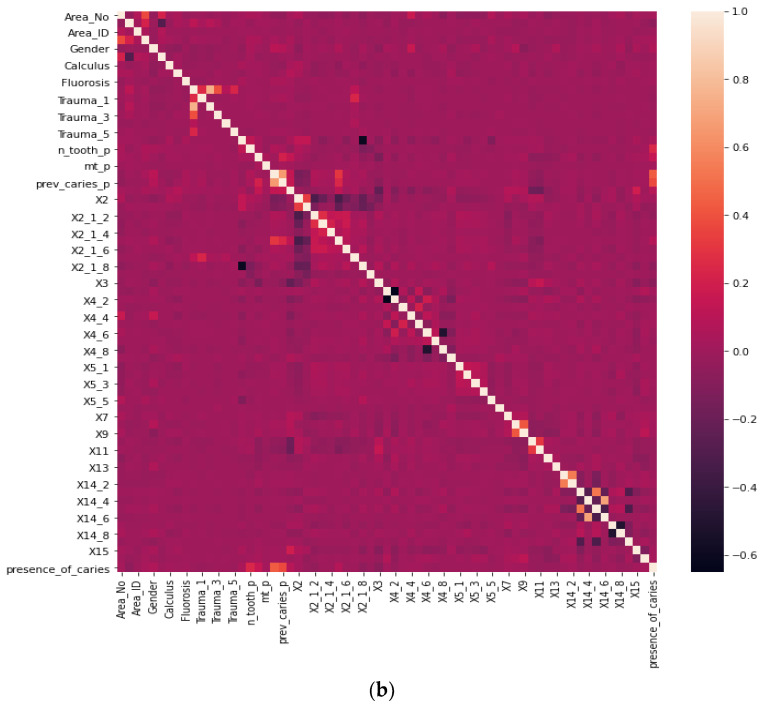
Dimensionality reduction by feature selection using Pearson Correlation method. (**a**) Before Pearson correlation for dimensionality reduction. (**b**) After applying Pearson correlation for dimensionality reduction.

**Figure 3:**
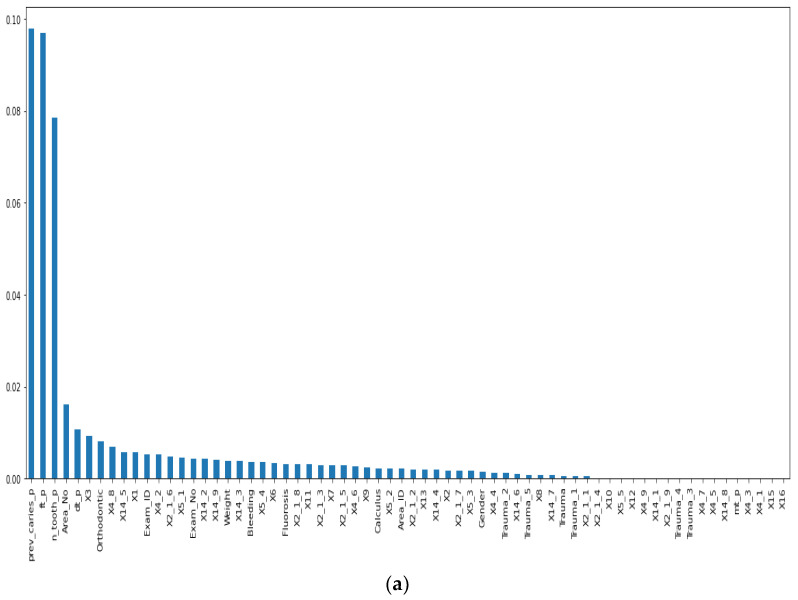

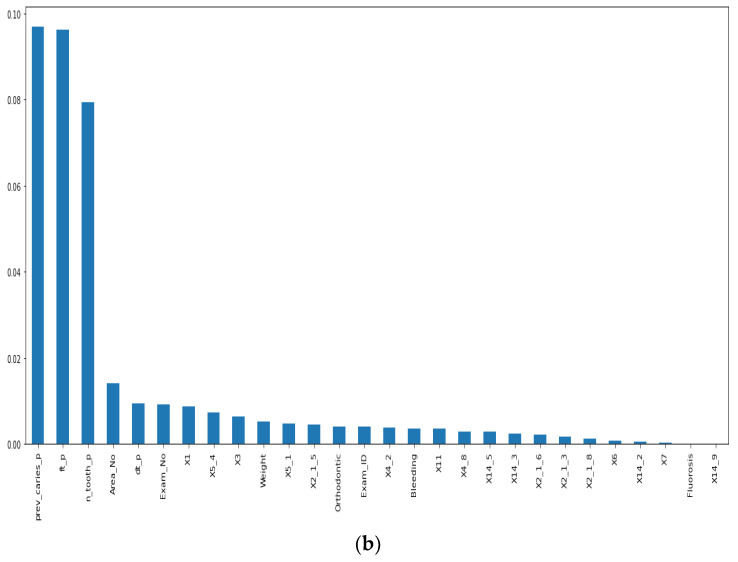
Dimensionality reduction by feature selection using Mutual information method. (**a**) Before applying mutual information to reduce dimensionality. (**b**) After mutual information method to reduce dimensionality.

**Figure 4:**
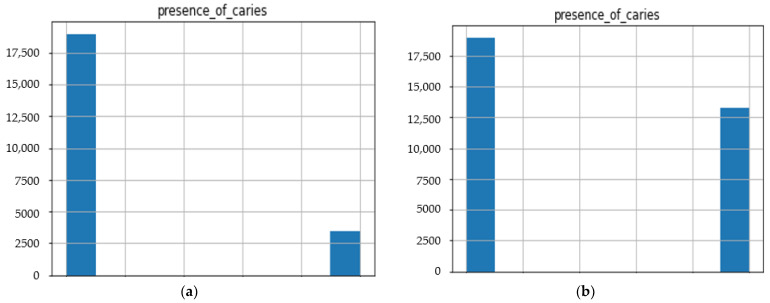
Data distribution ratio with respect to the target variable before and after SMOTE. (**a**) Data distribution of numerical dataset before SMOTE. (**b**) Data distribution of the numerical dataset after SMOTE.

Before our experiment, we manually cleaned our dataset by dropping unnecessary attributes after discussing them with medical doctors. During our experiments, we first performed feature selection (using Pearson correlation and Mutual information techniques) to remove irrelevant features, reduce the dataset’s dimensionality, therefore avoiding the curse of dimensionality, and keep the essential features to the target variable. The feature selection by Pearson Correlation selects features with less linear dependence and drops those with high correlation. We selected a threshold of 80% and dropped features with a correlation above that cusp value. Therein drops a total of ten features. Figure 2 shows the dataset dimensionality before and after Pearson correlation was applied. Then we used mutual information on the subset of features obtained from the Pearson correlation to further select features with higher dependency on the target label, therefore assessing the relevancy of the selected subset of features to the target output variable [36]. The mutual information relies on nonparametric methods based on entropy and measures the amount of data from one random variable given another. Figure 3 displays the dimension reduction performed on the dataset by applying mutual information. Secondly, it is essential to mention that the dataset was significantly unbalanced; the proportion of samples with label “0” (without caries) was more significant than the one with label “1” (with caries). This study uses Synthetic Minority Oversampling Technique (SMOTE) to handle the imbalanced problem by generating new data instances from samples of the feature space of each target class combined with the feature of its nearest neighbors [37]. Before the SMOTE method, the distribution ratio of the data was 92.40% (18,954) and 7.6% (3417) for labels “0” and “1”, respectively. After applying the SMOTE oversampling technique to the data, we obtained a ratio of 58.82% (18,954) for label “0” and 41.18% (13,267) for label “1”. Figure 4 shows the distribution ratio of the data sample before and after the SMOTE is applied. Once the data was balanced, we scaled our data sample to normalize the range of independent features, standardizing the input variables’ functionality range, obtaining a mean of 0 and a standard deviation of 1.

#### 3.2.2. Image Dataset Preparation

This work used imaging data to train on the second branch of the proposed algorithm. Before the training stage, some preprocessing had to be performed on this image data. During the data preprocessing step, we performed: image augmentation, resizing and normalization. Given the shortage of dental images online, using the collected image, we auto generated a considerable number of synthesized images via six geometric augmentation techniques (rotation, brightness, zoom, shear, width shift and height shift) with various factors (30, (range 0.1 to 1.5), 0.3, 0.1, 0.2 and 0.2 respectively). The image augmentation generated a ratio of 18,954 image samples for dental images without caries and 13,267 for dental with caries. Then image resizing was performed to uniformize the size differences observed in the samples and standardized all the image sizes to 32 × 32 pixels. This process has helped reduce the instability related to image size differences and resolve the memory leak faced. The last step of our image preprocessing was normalization, which is a process that consists of changing the range of pixel intensity values, ensuring a similar data distribution in each input parameter (pixel). Normalization of image samples is performed by subtracting the mean from each pixel, then dividing by the standard deviation. While keeping the pixel number positive, we chose the normalized scale of data to be in the range [0, 1].

### 3.3. Prediction Model

The MMDCP model was applied to the multi-modal data to predict the presence or absence of dental caries in patients. The proposed model fuses the features from both modalities (numerical dataset generated from hospital survey and the image dataset collected from the internet) and aggregates the collected information to increase the prediction accuracy. Among the different DL model available, this study combines two efficient methods suitable for the types of data used: ANN and DenseNet201. Figure 1 displays the conceptual representation of the MMDCP model.

The neural network structures of the MMDCP model separately extracted features from the two modalities and performed an intermediate-feature-level fusion. The ANN model extract features from the numerical dataset, while the DenseNet201 is applied to extract features from the imaging dataset. The fusion of the intermediate features was performed by concatenating the output vectors of the two DL models, and these generated output vectors are then passed to the classification layer to predict the state of patient oral health (presence of caries, absence of caries).

#### 3.3.1. Numerical Classifier

The standard ANN (Artificial Neural Network) algorithm was previously experimented on for the numerical dataset during our previously published work on dental caries [10]. As described in the data preprocessing section, the preprocessing step was performed to clean and select the essential features necessary for better performance. The selected features were sent as input to the ANN algorithm. The ANN algorithm used consisted of six dense layers, each followed by a dropout layer to prevent overfitting, and a flattened layer of four dimensions was added at the final layer. The input layer of the model was set to 28 dimensions, representing the 28 most essential features selected from the dataset. The ANN output is a set of intermediate features vectors that will be passed to the concatenation layer at the fusing stage for further operation. The ANN output vector is obtained from Equation (1).

(1) $$Z_{i}^{\left(n\right)}=\sum_{j=0;i,n=1}^{i,j,n}\left(\left(W_{10}^{\left(1\right)}a_{0}+b_{1}^{\left(1\right)}\right)+\left(W_{11}^{\left(1\right)}a_{1}+b_{1}^{\left(1\right)}\right)+\ldots +W_{ij}^{\left(n-1\right)}a_{j}+b_{i}^{\left(n-1\right)}\right)$$

(2) $$y_{ou\_ANN}=\sum_{k=1}^{k,L}Z_{k}^{\left(L\right)}$$

where $W_{ij}^{\left(n\right)}a_{j}$ represents the weight of the n layer between node i in the next layer and node j in the current layer. $b_{i}^{\left(n-1\right)}$ represent the bias introduced at layer n−1. $a_{j}$, the function of input at node j where the inputs at layer 1 [$a_{0},\;a_{1},\;a_{2},\;a_{3},\;\ldots ,\;a_{j}$] were the selected dataset features [age, gender, bleeding gum, …, calculus]. Equation (1) is the output of the node i at layer n. Equation (2) shows the numerical dental caries dataset features output vector at layer L.

#### 3.3.2. Image Classifier

On the second branch of the proposed model, to train on images data, we made good use of the pre-trained DenseNet algorithm [38], with the particularity of having all nodes directly connected, allowing feature mapping propagation in all subsequent layers, alleviation of vanishing-gradient problem, and reduction of the number of parameters. In DenseNet, the concept of feedforward is applied where each layer forwards its input feature together with the features received from the preceding convolutional block.

Considering the preprocessed dental caries image input of the DenseNet to be $x_{0}$; unlike ResNet, DenseNet performs a concatenation operation between incoming feature maps and output feature maps instead of summing them together. Therefore, the equation at layer *l^th^*:

(3) $$y_{ou\_DenseNet}=F_{l}=H_{l}\left([x_{0},x_{1,\;\;\;.\;\;.\;\;.\;\;}\;x_{l-1}\right])$$

where the layers 0 to l−1 have as concatenation output feature maps $\left([x_{0},x_{1,\;.\;.\;.\;}\;x_{l-1}\right])$, and H(.) representing the nonlinear transformation function is composed of Bach normalization (to reduce the number of epochs required during training and stabilize the learning process), ReLu (serving as activation layer) and a Convolutional layer.

Batch Normalization is obtained as shown below:

(4) $$\mu_{B}=\frac{1}{n}\sum_{j=1}^{n}x_{j}$$

(5) $$\sigma_{B}^{2}=\frac{1}{n}\sum_{j=1}^{n}{\left(x_{j}-\mu_{B}\right)}^{2}$$

(6) $$\;\hat{x_{j}}=\frac{x_{j}-\mu_{B}}{\sqrt{\sigma_{B}^{2}+\varepsilon }}$$

(7) $$y_{j}=\;\gamma \hat{x_{j}}+\beta$$

where Equations (4) and (5) represent batch mean and batch variance, respectively. Equation (6) shows the normalization of layer inputs using the batch statistics calculated previously. Finally, Equation (7) presents the output of the layer after scale and shift operations are performed. γ and β are two parameters to be learned during training along with the original network parameters.

The growth rate *K*, another important feature of DenseNet helps in generalizing the *l^th^* layer as follows [39]:

(8) $$K^{\left[l\right]}=\left(K^{\left[0\right]}+k\left(l-1\right)\right)$$

where the number of channels is represented as $K^{\left[0\right]}$.

#### 3.3.3. Concatenation Layer

To tackle the dental caries prediction using multi-modal data, intermediate features generated by ANN and DenseNet201 algorithms are merged. The vectors generated by the two networks, Equations (2) and (3), are concatenated together to produce the output vector as shown in Equation (9) which in turn is the input of the classifier to predict the presence or absence of dental caries.

(9) $$Vout=W_{out\_c}\left(y_{out_{ANN}}\;;\;y_{ou\_DenseNet}\right)+b_{out\_c}$$

where $W_{out\_C}$ is a weight matrix, and $b_{out\_c}$ is the bias vector added to make the model less sensitive to some data points. Then the concatenate output vector Vout is pass to a binary classification layer to predict the presence or absence of dental caries.

## 4. Experimentation

### 4.1. Experimental Setup

With the complexity of the proposed model and the enormous dataset used, an optimal experimental environment was required for the success of our work. This study conducts the experiments on a computer with the following settings (clearly subdivided into two parts as shown in Table 1): 64-bit Ubuntu version 18.04 Operating System (OS), Intel Core i7-6850K at 3.60 GHz, NVIDIA GPU 1080 Ti GTX and 62.7 GB RAM. The model was constructed using TensorFlow, Python version 3.8.0, and a CUDA version 11.0.

### 4.2. Dataset

#### 4.2.1. Numerical Dataset

The first is a public dataset obtained from the Korean Center for Disease Control and Prevention under the Ministry of Health and Welfare [10]. After the survey, surveyors gathered 22,371 children’s oral health status and collected ninety different attributes from each child (age, gender, caries history, tooth bleeding, etc.). The participants were grouped into two groups, those with dental caries (labeled “1”) and those without caries (labeled as “0”). The distribution ratio of the whole dataset was 18,954 and 3417 samples for label “0” and label “1”, respectively.

#### 4.2.2. Image Dataset

**Figure 5:**
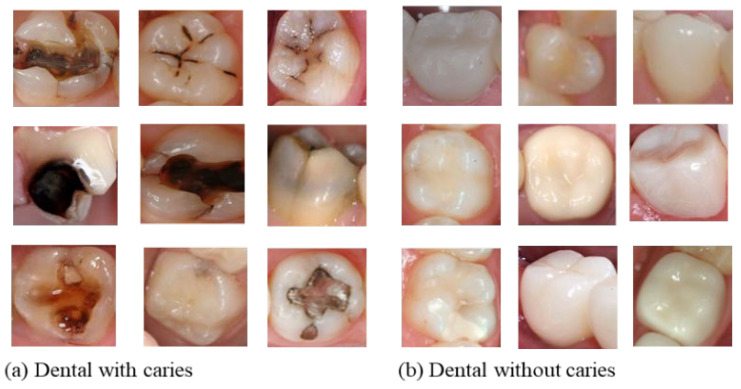
Dental image samples used during model training.

The second dataset used in this work was collected from various online sources [34,35] due to the unavailability of the public dataset on the dental image. The dataset constructed consisted of 32,221 image samples divided into two classes: 18,954 for the class labeled “0” (dental images without dental caries) and 13,267 for the category labeled “1” (dental images with dental caries). Given the difference present in images, we performed an image resizing during the preprocessing and uniformized the images to a size of 32 × 32 pixels. The following Figure 5 shows a few image samples of each class.

## 5. Result and Discussion

### 5.1. Metrics of Evaluation

During our experiments, to prevent overfitting while accurately predicting dental caries, each dataset was split into two, a training set (80% of the entire data set) and a test set (20% of the data set). Then the training set was further divided into training (80% of the training samples) and validation set (20% of the training samples). Given the nature of the task at hand, evaluation metrics (precision, recall, F1-score, accuracy, AUC-ROC) for classification problems were used to evaluate the performance of the proposed model, therefore observed to avoid the overfitting. The following Table 2 describes the used evaluation metrics in detail.

Where:

- *TP* (True Positive): positive observation predicted as positive;
- *TN* (True Negative): negative observation that was correctly predicted as being negative;
- *FP* (False Positive): negative observation wrongly predicted as positive;
- *FN* (False Negative): positive observation wrongly predicted as negative;
- *TPR* (True Positive Rate): is the percentage of class labeled as “0” (absence of caries) points incorrectly classified by the model;
- *FNR* (False Negative Rate): is the percentage of class labeled “1” (presence of caries) points correctly classified by the model.

### 5.2. Proposed Model Results

**Figure 6:**
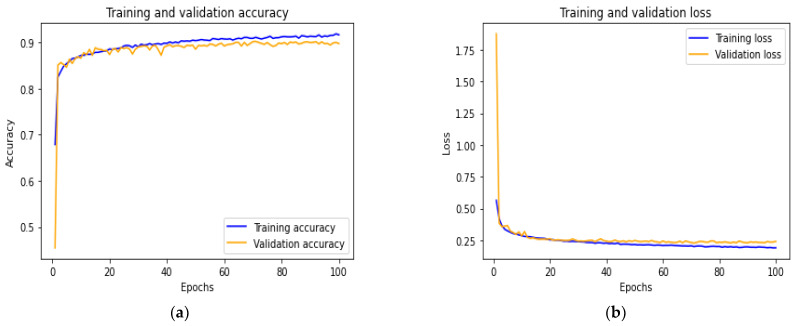
Accuracy vs. loss of the proposed model on training and validation set. (**a**) Accuracy of the training vs. validation set. (**b**) Loss of the model on training vs. validation set.

During our experiments, we used metadata gathered from the numerical and dental image datasets to predict the presence or absence of dental caries. Different features from the multi-modal dental datasets were extracted to train the hybrid model (constructed of ANN and DenseNet201) to predict dental caries. Our datasets were both divided into three sets: the training set of each modality is used on their respective network, allowing the hybrid model to identify a pattern within the data samples; the validation set is used to validate the model performance during training and fine-tuning the model hyperparameters; and, finally, the test set: used assess the performance of the trained hybrid model. The commonly used metrics of evaluation (accuracy, precision, recall, f1-score, AUC-ROC) for classification problems were selected to evaluate the proposed model. Figure 6 demonstrates the model performance on the training and validation set. Figure 6a shows the accuracy of training and validation, while Figure 6b displays the loss of progress on the training and validation set. These results show that overfitting was avoided through proper model layers settings. Table 3 displays the performance evaluation of the proposed model on the test set. From Table 3, we can observe the Macro average (which calculates the mean of the different binary metrics while giving equal weight to all the classes. It is necessary to monitor this metric if a class imbalance exists on the dataset used); weighted average (each metric is calculated with respect to the number of samples present in each class); support (is the number of samples present in each class of the test set used to evaluate the model prediction performance). Table 3 shows the proposed model prediction performances of 0.90, 0.89, 0.90 and 0.89 for accuracy, precision, recall and f1-score, respectively.

**Figure 7:**
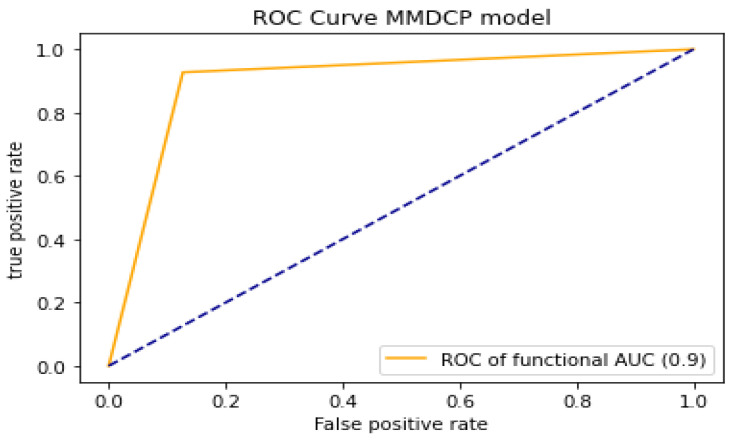
Performance evaluation of the proposed model using AUC-ROC.

To analyze and evaluate the model’s capability in distinguishing the different classes (label “0” absence of dental caries, label “1” presence of dental caries), we proceeded by computing the AUC-ROC of the model. The higher the AUC-ROC value, the better the model performance when distinguishing between the label “0” and label “1” classes. The proposed model yielded an AUC-ROC of 0.90. Figure 7 shows the performance evaluation of the proposed model in the case of AUC-ROC.

**Figure 8:**
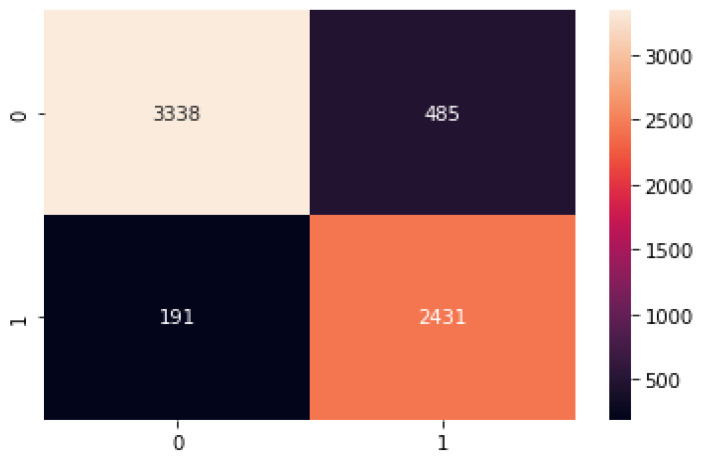
Confusion matric of the proposed model on the test set.

Finally, to summarize the performance of the proposed model by comparing the frequency of the predicted classes to the expected classes, we performed a confusion matrix of the proposed model, as shown in the Figure 8 below.

### 5.3. Discussion

In this study, we proposed a novel MMDCP model used to predict dental caries among young people living in Korea; one of the datasets was provided by the Korean Center for Disease Control and Prevention under the Ministry of Health and Welfare. The critical difference between this work’s novel approach and prior works is that it has leveraged the usage of multi-modal data for the training on a hybrid model (a form of an artificial neural network and a Dense Convolutional network). Using the classification metrics of evaluation to assess the model performance, the results obtained in this study show that the proposed multi-modal prediction model allows to accurately predict the presence or absence of dental caries in patients. This work displays the importance of multi-modality in developing prediction models on healthcare datasets. Consequently, future works should consider using several modalities to improve the training technique’s performance.

From the thorough analysis performed to assess the novel model performance in significantly predicting dental caries, the multi-modal dental caries prediction model proposed in this work yielded more robust prediction results than some previously published work on dental caries prediction using a single modality dataset. As such, the authors of [40] presented a paper on multifactorial modeling intending to predict caries increment in an adolescent population; during their experiments, the authors obtained an accuracy slightly below 80%, which was 10% lower than the novel model proposed in this study. Ref. [14] proposed a Generalized regression neural network model for dental caries prediction among the Geriatric residents of Liaoning (China). This study yielded an accuracy, sensitivity, specificity, and AUC-ROC values of 77.29%, 85.16%, 70.27% and 0.626, respectively, while our model displayed higher values, as described in the model evaluation result section. The authors of [41] published a novel model to predict the risk of caries among 1055 teenagers using ML algorithm (random forest (RF) and logistic regression (LR)). While experimenting with the training set and evaluating their models on the test set, they obtained an AUC-ROC of 0.74 for LR and 0.73 for RF.

Regardless of the impressive results generated by the prior published works on dental caries prediction, the novel approach present in this study displays significant results showing the importance of multi-modality and hybrid networks for prediction operation.

To the best of our knowledge, this study conducted the first study using a multi-modal dataset (numerical and imaging) for dental caries prediction using the DL technique, and we firmly believe that using multi-modality in oral healthcare can be a big step toward the development of more efficient prediction models in dental caries prediction.

## 6. Conclusions

In this study, we introduced a novel method to predict dental caries using a multi-modal dataset (applied to a hybrid neural network model) that combines numerical and image data. In order to perform a binary classification and predict the presence or absence of dental caries, the model was fed the extracted features from the two modalities. Numerous experiments were performed on the datasets, and the results revealed that the proposed model was very effective. When compared to numerous earlier single modality models for the prediction of dental caries, the model presented in this work displayed better accuracy, precision, recall, f1-score, and AUC-ROC. As a result, this highlights the high likelihood of using multi-modality in dental caries prediction.

During the experimentation stage, there were some limitations that had to be overcome, such as the lack of a large collection of X-ray dental images, by using dental images and data augmentation as described in the data preparation section. For our upcoming work, the labeling of X-ray images dataset is currently being performed.

The suggested novel approach performed admirably when evaluated using the test data and could thus be applied to the development of a diagnosis tool that can aid dentists in accurately and meaningfully recommending treatments to patients who have a past, present, or future risk of developing dental caries. In order to improve oral healthcare and lessen the burden on the population, we are currently working on the implementation of a decision support system for dental caries prediction.

## Figures and Tables

**Table 1:** Experimental setup environment.

Part	Component	Description
Hardware	OS	Ubuntu 18.0.4 64 bit
	CPU	Intel Core i7-6850k (3.60 Hz)
	GPU	NVIDIA GTX 1080 Ti
	RAM	62.7 GB
Software	TensorFlow	2.5.0
	Python Version	3.8.0
	CUDA Version	11.2.0

**Table 2:** Performance evaluation metric of the proposed model.

Metric	Formula	Description
Precision	$\frac{TP}{TP+FP}$	Indicates the proportion of positive identifications which were correct.
Recall	$\frac{TP}{TP+FN}$	Indicates the proportion of actual positives which were correctly classified
F1-score	$2\times \frac{\left(Precision\times Recall\right)}{\left(Precision+Recall\right)}$	Combination of precision and recall
Accuracy	$\frac{TP+FP}{\left(TN+TP+FN+FP\right)}$	Overall performance of the model
AUC-ROC	$\frac{1+TPR-FPR}{2}$	Comparison of a model’s TPR versus model’s FPR

**Table 3:** Classification report of the proposed hybrid model on the test set.

	Precision	Recall	F1-Score	Support
0	0.95	0.87	0.91	3823
1	0.83	0.93	0.88	2622
Accuracy			0.90	6445
Macro average	0.89	0.90	0.89	6445
Weight average	0.90	0.90	0.90	6445

## Data Availability

The data supporting the findings of this study are not publicly available and can be made available from the corresponding author upon reasonable request. The source code is publicly available at: https://github.com/Soualihou237/MMDCP-Multi-modal-dental-caries-prediction (accessed on 11 July 2022).

## References

[1] US Surgeon General Report (2000). Oral health in America: A report of the Surgeon General. J. Calif. Dent. Assoc..

[2] Haworth S., Dudding T., Waylen A., Thomas S.J., Timpson N.J. (2017). Ten years on: Is dental general anesthesia in childhood a risk factor for caries and anxiety?. Br. Dent. J..

[3] Foster T., Perinpanayagam H., Pfaffenbach A., Certo M. (2006). Recurrence of early childhood caries after comprehensive treatment with general anesthesia and follow-up. J. Dent. Child.

[4] Bowen W.H., Birkhed D., Granath L., McHugh W.D. (1986). Dental caries: Dietary and microbiology factors. Systemized Prevention of Oral Disease: Theory and Practice.

[5] Stephan R.M. (1944). Intra-oral hydrogen-ion concentrations associated with dental caries activity. J. Dent. Res..

[6] Weiss R.L., Trithart A.H. (1960). Between-meal eating habits and dental caries experience in preschool children. Am. J. Public Health Nation’s Health.

[7] Black G.V. (1916). Mottled teeth: An endemic developmental imperfection of the enamel of the teeth heretofore unknown in the literature of dentistry. Dent. Cosm..

[8] Zero D.T., Fontana M., Martínez-Mier E.A., Ferreira-Zandoná A., Ando M., González-Cabezas C., Bayne S. (2009). The Biology, Prevention, Diagnosis and Treatment of Dental Caries. J. Am. Dent. Assoc..

[9] Hung M., Voss M.W., Rosales M.N., Li W., Su W., Xu J., Bounsanga J., Ruiz-Negrón B., Lauren E., Licari F.W. (2019). Application of machine learning for diagnostic prediction of root caries. Gerodontology.

[10] Kang I.-A., Ngnamsie Njimbouom S., Lee K.-O., Kim J.-D. (2022). DCP: Prediction of Dental Caries Using Machine Learning in Personalized Medicine. Appl. Sci..

[11] Richens J.G., Lee C.M., Johri S. (2020). Improving the accuracy of medical diagnosis with causal machine learning. Nat. Commun..

[12] Baiju R.M., Peter E., Varghese N.O., Sivaram R. (2017). Oral Health and Quality of Life: Current Concepts. J. Clin. Diagn. Res..

[13] Liu L., Wu W., Zhang S.-Y., Zhang K.-Q., Li J., Liu Y., Yin Z.-H. (2020). Dental Caries Prediction Based on a Survey of the Oral Health Epidemiology among the Geriatric Residents of Liaoning, China. BioMed Res. Int..

[14] Zaorska K., Szczapa T., Borysewicz-Lewicka M., Nowicki M., Gerreth K. (2021). Prediction of Early Childhood Caries Based on Single Nucleotide Polymorphisms Using Neural Networks. Genes.

[15] Kallenberg M., Petersen K., Nielsen M., Ng A.Y., Diao P., Igel C., Vachon C.M., Holland K., Winkel R.R., Karssemeijer N. (2016). Unsupervised Deep Learning Applied to Breast Density Segmentation and Mammographic Risk Scoring. IEEE Trans. Med. Imaging.

[16] Esteva A., Kuprel B., Novoa R.A., Ko J., Swetter S.M., Blau H.M., Thrun S. (2017). Dermatologist-level classification of skin cancer with deep neural networks. Nature.

[17] Hannun A.Y., Rajpurkar P., Haghpanahi M., Tison G.H., Bourn C., Turakhia M.P., Ng A.Y. (2019). Cardiologist-level arrhythmia detection and classification in ambulatory electrocardiograms using a deep neural network. Nat. Med..

[18] Nabilla M., Brenda C., Ichwan S.J.A., Arief C. (2021). Deep learning convolutional neural network algorithms for the early detection and diagnosis of dental caries on periapical radiographs: A systematic review. Imag. Sci. Dent..

[19] Lee J.H., Kim D.H., Jeong S.N., Choi S.H. (2018). Detection, and diagnosis of dental caries using a deep learning-based convolutional neural network algorithm. J. Dent..

[20] Casalegno F., Newton T., Daher R., Abdelaziz M., Lodi-Rizzini A., Schürmann F., Krejci I., Markram H. (2019). Caries detection with near-infrared transillumination using deep learning. J. Dent. Res..

[21] Zanella-Calzada L.A., Galván-Tejada C.E., Chávez-Lamas N.M., Rivas-Gutierrez J., Magallanes-Quintanar R., Celaya-Padilla J.M., Galván-Tejada J.I., Gamboa-Rosales H. (2018). Deep artificial neural networks for the diagnostic of caries using socioeconomic and nutritional features as determinants: Data from NHANES 2013-2014. Bioengineering.

[22] Lee S., Oh S.I., Jo J., Kang S., Shin Y., Park J.W. (2021). Deep learning for early dental caries detection in bitewing radiographs. Sci. Rep..

[23] Zheng L., Wang H., Mei L., Chen Q., Zhang Y., Zhang H. (2021). Artificial intelligence in digital cariology: A new tool for the diagnosis of deep caries and pulpitis using convolutional neural networks. Ann. Transl. Med..

[24] Zhang X., Liang Y., Li W., Liu C., Gu D., Sun W., Miao L. (2022). Development and evaluation of deep learning for screening dental caries from oral photographs. Oral Dis..

[25] Li W., Zhang Z., Song A. (2021). Physiological-signal-based emotion recognition: An odyssey from methodology to philosophy. Measurement.

[26] Chakraborty S., Aich S., Joo M., Sain M., Kim H.-C. (2019). A Multichannel Convolutional Neural Network Architecture for the Detection of the State of Mind Using Physiological Signals from Wearable Devices. J. Healthc. Eng..

[27] Bota P., Wang C., Fred A., Silva H. (2020). Emotion Assessment Using Feature Fusion and Decision Fusion Classification Based on Physiological Data: Are We There Yet?. Sensors.

[28] Murugappan R., Bosco J.J., Eswaran K., Vijay P., Vijayaraghavan V. User Independent Human Stress Detection. Proceedings of the 2020 IEEE 10th International Conference on Intelligent Systems (IS).

[29] Uddin M.T., Canavan S. Synthesizing Physiological and Motion Data for Stress and Meditation Detection. Proceedings of the 2019 8th International Conference on Affective Computing and Intelligent Interaction Workshops and Demos (ACIIW).

[30] Venugopalan J., Tong L., Hassanzadeh H.R., Wang M.D. (2021). Multimodal deep learning models for early detection of Alzheimer’s disease stage. Sci. Rep..

[31] Pingali L. Personal Oral Health Advisor Using Multimodal Sensing and Machine Learning with Smartphones and Cloud Computing. Proceedings of the 2019 IEEE International Conference on Cloud Computing in Emerging Markets (CCEM).

[32] Tiulpin A., Klein S., Bierma-Zeinstra S.M.A., Thevenot J., Rahtu E., Meurs J.V., Oei E.H.G., Saarakkala S. (2019). Multimodal Machine Learning-based Knee Osteoarthritis Progression Prediction from Plain Radiographs and Clinical Data. Sci. Rep..

[33] Nie D., Zhang H., Adeli E., Liu L., Shen D. (2016). 3D Deep Learning for Multi-modal Imaging-Guided Survival Time Prediction of Brain Tumor Patients. Med. Image Comput. Comput. Assist. Interv..

[34] Teeth_Dataset|Kaggle. https://www.kaggle.com/datasets/pushkar34/teeth-dataset.

[35] Teethdecay|Kaggle. https://www.kaggle.com/datasets/snginh/teethdecay.

[36] Beraha M., Metelli A.M., Papini M., Tirinzoni A., Restelli M. Feature Selection via Mutual Information: New Theoretical Insights. Proceedings of the International Joint Conference on Neural Networks (IJCNN).

[37] Chawla N.V., Bowyer K.W., Hall L.O., Kegelmeyer W.P. (2002). SMOTE: Synthetic Minority Over-sampling Technique. J. Artif. Intell. Res..

[38] Huang Z., Zhu X., Ding M., Zhang X. (2020). Medical Image Classification Using a Light-Weighted Hybrid Neural Network Based on PCANet and DenseNet. IEEE Access.

[39] Hasan N., Bao Y., Shawon A., Huang Y. (2021). DenseNet Convolutional Neural Networks Application for Predicting COVID-19 Using CT Image. SN Comput. Sci..

[40] Raitio M., Pienihäkkinen K., Scheinin A. (1996). Multifactorial modeling for prediction of caries increment in adolescents. Acta Odontol. Scand..

[41] Pang L., Wang K., Tao Y., Zhi Q., Zhang J., Lin H. (2021). A New Model for Caries Risk Prediction in Teenagers Using a Machine Learning Algorithm Based on Environmental and Genetic Factors. Front. Genet..

